# Clinical- and cost-effectiveness of a technology-supported and solution-focused intervention (DIALOG+) in treatment of patients with chronic depression—study protocol for a multi-site, cluster randomised controlled trial [TACK]

**DOI:** 10.1186/s13063-022-06181-4

**Published:** 2022-03-28

**Authors:** Philip McNamee, Aleksandra Matanov, Lauren Jerome, Sally Kerry, Neil Walker, Yan Feng, Andrew Molodynski, Shonagh Scott, Latha Guruvaiah, Sue Collinson, Rose McCabe, John Geddes, Stefan Priebe, Victoria Bird

**Affiliations:** 1 Unit for Social and Community Psychiatry, Newham Centre for Mental Health, Cherry Tree Way, London, S13 8SP UK; 2 Pragmatic Clinical Trials Unit, Centre for Evaluation and Methods, Queen Mary University, London, Yvonne Carter Building, 58 Turner Street, London, E1 2AB UK; 3 Oxford Health NHS Foundation Trust, Research & Development, Warneford Lane, Headington, Oxford, OX3 7JX UK; 4 Sheffield Health and Social Care NHS Foundation Trust, Research & Development, Fulwood House, Old Fulwood Road, Sheffield, S10 3TH UK; 5 Gloucestershire Health and Care NHS Foundation Trust, Research & Development, Fritchie Centre, Charlton Lane, Cheltenham, GL53 9DZ UK; 6 Homerton University Hospital, Homerton Row, London, E9 6SR UK; 7 Centre for Mental Health Research, City University of London, Northampton Square, London, EC1V 0HB UK; 8 Department of Psychiatry, University of Oxford, Warneford Hospital, Warneford Lane, Headington, Oxford, OX3 7JX UK

**Keywords:** Cluster randomised trial, Depression, Community care, Mental health, Solution focused

## Abstract

**Background:**

Many with an acute depressive disorder go on to develop chronic depression, despite ongoing care. There are few specifically designed interventions to treat chronic depression. DIALOG+, a technology-assisted intervention based on the principles of solution-focused therapy, may be beneficial. It has been shown to be effective as a treatment for patients with psychotic disorders, especially in regards to increasing quality of life. DIALOG+ was designed to be flexibly applied and not diagnosis-specific, aiming to structure communication and generate a personally-tailored care plan. This cluster randomised controlled trial (RCT) is part of a programme of research to adapt and test DIALOG+ for patients with chronic depression.

**Methods:**

Patients will be eligible for the trial, if they have exhibited symptoms of depression or non-psychotic low mood for at least 2 years, have regular contact with a clinician and have a low subjective quality of life and moderate depressive symptoms. Clinicians, who routinely see eligible patients, will be recruited from a number of sites across NHS England. Clusters will have between 1 and 6 patients per clinician and will be randomised in a 1:1 ratio to either the intervention (DIALOG+) or active control group (treatment as usual + DIALOG scale). Clinicians in the intervention group are trained and asked to deliver the intervention regularly for 12 months. Active control participants receive treatment as usual and are asked to rate their satisfaction with areas of life and treatment on the DIALOG scale at the end of the clinical session. Approximately 112 clinician clusters will be recruited to reach a total patient sample size of 376. Clinical and social outcomes including costs are assessed at baseline and 3, 6 and 12 months post randomisation. The primary outcome will be subjective quality of life at 12 months.

**Discussion:**

This definitive multi-site, cluster RCT aims to evaluate the clinical- and cost-effectiveness of DIALOG+ for people with chronic depression. If shown to be effective for this patient population it could be used to improve outcomes of mental health care on a larger scale, ensuring that patients with complex and co-morbid diagnoses can benefit.

**Trial registration:**

ISRCTN11301686. Registered on 13 Jun 2019.

## Administrative information

**Table:** 

Title {1}	Clinical- and cost-effectiveness of a technology supported and solution-focused intervention (DIALOG+) in treatment of patients with chronic depression – study protocol for a multi-site, cluster randomised controlled trial [TACK]
Trial registration {2a and 2b}.	ISRCTN11301686; 10.1186/ISRCTN11301686
Protocol version {3}	Study Protocol v9.0, 30.Sep.2022
Funding {4}	The trial makes up part of a programme grant funded by the National Institute of Health Research (NIHR), Programme Grants for Applied Research (PGfAR; RP-PG-0615-200010)
Author details {5a}	a. Philip McNamee, Aleksandra Matanov, Lauren Jerome, Stefan Priebe & Victoria Bird- Unit for Social and Community Psychiatry, Queen Mary University London b. Sally Kerry, Neil Walker, Yan Feng- Pragmatic Clinical Trials Unit, Queen Mary University of London c. Andrew Molodynski- Oxford Health NHS Foundation Trust d. Shonagh Scott- Sheffield Health and Social Care NHS Foundation Trust e. Latha Guruvaiah- Gloucestershire Health and Care NHS Foundation Trust f. Sue Collinson- Homerton University Hospital g. Rose McCabe- Centre for Mental Health Research, City University of London h. John Geddes- Department of Psychiatry, University of Oxford
Name and contact information for the trial sponsor {5b}	Noclor is the sponsor of the trial on behalf of East London NHS Foundation Trust. Address: 1st Floor, Bloomsbury Building St Pancras Hospital, 4 St Pancras Way, London, NW1 0PE Tel: 020 7685 5949 E: sponsor.noclor@nhs.net
Role of sponsor {5c}	The sponsor has the responsibility for proportionate and effective arrangements being in place to set up, run and report the research project to a high standard that meets the requirements of good clinical practice.

## Introduction

### Background and rationale

The effective treatment of depression is a priority within the NHS [1], not just because of its relatively high prevalence, but also because it is a leading cause of disability worldwide [2]. The economic burden on the NHS and wider society is high, due to patients being high utilisers of healthcare services as well as experiencing work productivity impairments [3]. Despite existing evidence-based interventions aiming to reduce the impact of depression, there has been no reduction in the global prevalence or burden of depression since 1990 [4], and the number of people experiencing depression within the UK is set to increase to 1.45 million by 2026 [5].

Furthermore, over a third of people who experience an acute episode of depression do not adequately improve and instead go on to develop a chronic disorder, often labelled ‘treatment resistant’ [6]. Chronic depression is associated with poor clinical and social outcomes including an increased suicide risk, poor quality of life, physical comorbidity, reduced social networks and functional impairment [5–7]. Chronic depression is broadly defined as 2 years of continuous symptoms in individuals with mood disorder [8]. Past research has tended to focus on the treatment of episodic depression, resulting in a lack of evidence-based interventions specifically tailored for chronic forms [9]. Chronic depression is linked with worse socioeconomic and interpersonal conditions than episodic depression [10, 11] and large numbers of chronically depressed patients do not receive appropriate treatment [12]. Many patients with chronic depression in the UK are managed in secondary mental health services and receive treatment from clinicians from a range of fields (e.g. psychiatrists, mental health nurses, social workers, support workers) known as a care coordinator. Care coordination involves regularly meeting with a named mental health professional to co-ordinate the assessment and planning of their care, including regular reviews. However, these meetings are not founded on evidence-based methods to improve outcomes and vary widely between sites [13]. Furthermore, established pharmacological and psychological treatments, such as antidepressants or psychotherapy have at best only limited efficacy for this patient group [14]. Consequently, there is a need to develop interventions that are both clinically and cost-effective which can be routinely implemented within different clinical settings to make routine care more effective in improving patient outcomes.

DIALOG+, a technology-assisted and resource-oriented intervention, represents one possible treatment solution. This intervention structures communication between patients and their clinicians during routine meetings in mental health care settings, aiming to create better treatment plans and improve clinical outcomes. DIALOG+ consists of a patient-centred assessment (containing 8 quality of life areas and 3 treatment aspects) whereby patients rate their satisfaction with these 11 different areas of life and treatment, on a tablet computer. These routinely collected scores can then be integrated into the discussion between clinician and patient, and used to compare ratings between different areas in the same session, or across the same area over time. The ratings are also used to select up to 3 of the areas for more detailed discussions. This discussion is guided by a 4-step approach, informed by the principles of brief solution-focused therapy. The effectiveness of DIALOG+ was previously established for patients with psychosis treated in the community [15]. A single site, cluster randomised controlled trial with this population found that patients who used the intervention over 6 months had improved quality of life, fewer unmet needs, lower general symptom levels, better social outcomes and lower NHS treatment costs [16].

Previous research has indicated that patients with chronic depression typically have an even lower quality of life compared to those with psychotic disorders [17], meaning that an intervention like DIALOG+ which targets quality of life has increased scope to improve satisfaction and recovery. There is an emerging evidence base from the application of DIALOG+ in small controlled trials in lower middle-income countries (LMICs) [18, 19] that the intervention is suitable and effective in those with depression. However, patients involved in these trials had less chronic forms of depression, and a definitive, and amply powered trial is required to test the clinical and cost-effectiveness of DIALOG+ in improving treatment outcomes of patients with chronic depression.

The trial makes up a substantial part of the “Tackling Chronic Depression” (TACK) Programme Grant (RP-PG-0615-20010), which is funded by the National Institute of Health Research (NIHR) through the Programme Grant funding stream. The funder had no role in the design of the study nor the data collection or analysis. The overall aim of the programme is to adapt DIALOG+ to the needs of patients with chronic depression and test its effectiveness. The views expressed are those of the author(s) and not necessarily those of the NIHR or the Department of Health and Social Care.

Following earlier exploratory work where clinicians and patients tested the use of DIALOG+ in routine sessions for a 3-month period, and were then interviewed about their experiences, found the basics of the intervention needed no fundamental changes to make it appropriate for this specific patient population [20]. This was followed by a multi-site feasibility randomised controlled trial (*in prep*) which demonstrated that the intervention was acceptable and feasible and that the trial procedures were appropriate.

### Objectives

The primary objective of this definitive trial is to establish whether the regular use of DIALOG+ over a 12-month period, in various clinical settings, can improve quality of life in patients with chronic depression, compared with an active control.

Secondary objectives are:

- To evaluate whether the intervention improves secondary outcomes such as depression symptom severity, treatment satisfaction, and health-related quality of life.
- To assess the costs of intervention delivery and to establish the cost-effectiveness of the intervention.
- To explore the implementation of the intervention, particularly in regards to clinician training requirements and fidelity to the manual.

### Trial design

A pragmatic cluster-randomised controlled trial design will be used to test the study objectives. Clinicians, and their patients (who together form a cluster), will be randomly allocated to either the experimental (DIALOG+) group or to an active control group (treatment as usual (TAU) + DIALOG scale). Clinicians will act as the unit of randomisation, with clustering by clinician to prevent contamination effects within the study. Clinicians allocated to the experimental arm will use DIALOG+ to structure their routine sessions over a 12-month period. Clinicians allocated to the active control arm will deliver routine care but additionally ask the patient to complete the 11-item DIALOG scale on a tablet computer at the end of every session, but without any clinical input or discussion of the items.

In both arms, the interventions will be delivered within the context of routine care and therefore will be delivered wherever or however these routine meetings usually take place. This could be within community mental health services, outpatient clinics, GP surgeries and/or at the patient’s home, or delivered remotely over the phone or on NHS Trust-approved web-conferencing platforms (e.g. MS Teams). No additional sessions or clinician time will be required to deliver the intervention.

Clinicians will be recruited first, and will then identify eligible patients from their caseloads. Cluster sizes will range from a minimum of 1 to a maximum of 6 patients, with an average cluster size between 3 and 4. Randomisation will take place once the cluster is complete- either when the maximum cluster size is reached or when no more eligible participants can be identified from the clinician’s caseload.

Clinicians will use the intervention, with each patient, monthly (on average) for the first 6 months with additional sessions during the following 6 months (e.g. at 8 and 10 months) at the clinician’s discretion.

There will be four data collection points: baseline, 3, 6 and 12 months after the date of randomisation (see Table 1).

**Table 1:**
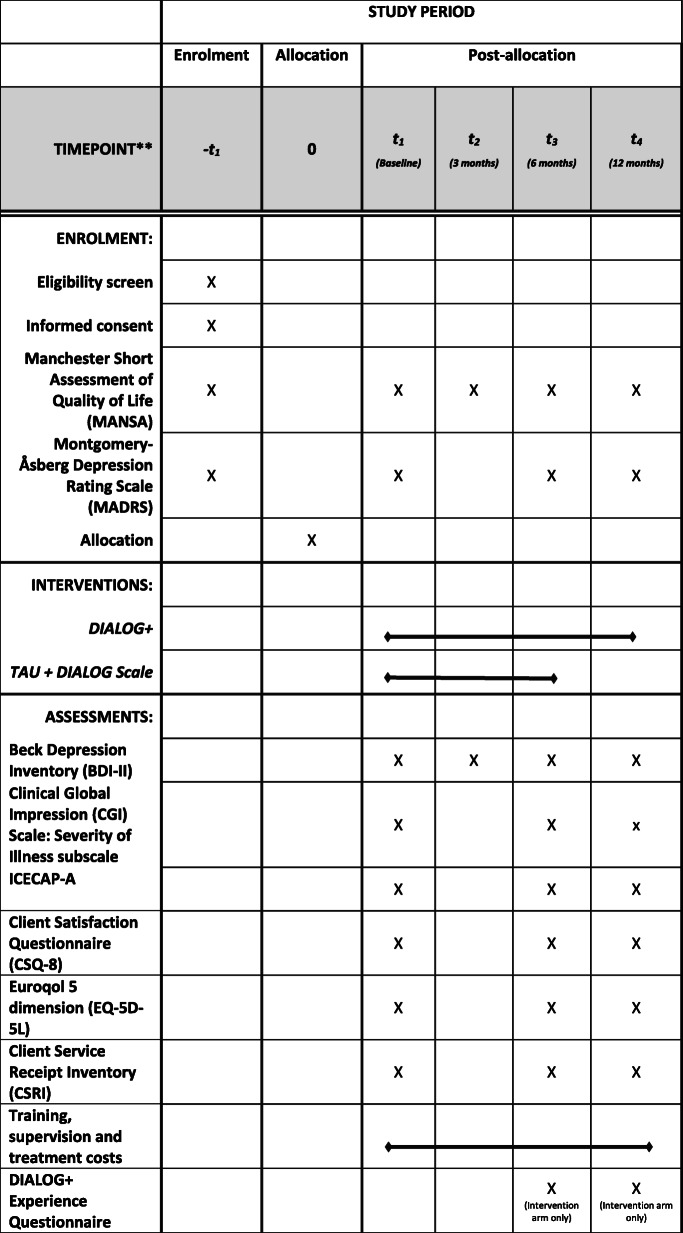
SPIRT figure outlining schedule of enrolment, interventions and assessments

An internal pilot was conducted within three of the trial sites (Oxford Health NHS Foundation Trust, Gloucestershire Health & Social Care NHS Foundation Trust and Sheffield Health and Social Care NHS Foundation Trust) during the first 4 months of the trial. Stop-Go criteria were developed *a priori* based on recruitment rates and clinician training rates. The trial was launched on the 26th of June 2019, and criteria for continuation of the trial were met according to the Programme Steering Committee. Data collected as part of the internal pilot will be analysed alongside all other trial data.

The trial is supported by the Pragmatic Clinical Trial Unit (PCTU), based at Queen Mary University of London.

The SPIRIT Reporting guidelines [21] were used to structure this protocol. The completed SPIRIT figure can be found at Table 1. The full SPIRIT checklist can be found as an additional file.

## Methods: Participants, interventions and outcomes

### Study setting

This multi-centre study will be coordinated by the East London NHS Foundation Trust (ELFT), based at the Newham Centre for Mental Health. Trial sites, all of which will be NHS England mental health trusts, will be purposefully selected based on eligible patient numbers and to represent a mix of urban, semi-urban and rural areas to allow for variation in demographics amongst the sample (a list of current sites can be seen in Table 2). Multi-disciplinary staff from community mental health teams (CMHTs), including Older Adult services, as well as intermediary and primary care services (where available within secondary care Trusts), will be approached for inclusion in the study.

**Table 2:** List of trial sites

Gloucestershire Health & Social Care NHS Foundation Trust Oxford Health NHS Foundation Trust Sheffield Health and Social Care NHS Foundation Trust Devon Partnership NHS Foundation Trust Essex Partnership NHS Foundation Trust Somerset NHS Foundation Trust North East London NHS Foundation Trust Lancashire and South Cumbria NHS Foundation Trust South London and Maudsley NHS Trust	

The study was designed with complexity and diversity in mind, given both the variability of care coordination practices across the UK [13] and the differing definitions of chronic depression [7]. The study team adopted an inclusive approach in the design of the trial, particularly in the eligibility criteria, to ensure the trial was pragmatic as possible and reflected clinical reality in the treatment of chronic depression within the UK.

### Eligibility criteria

To reflect the pragmatic nature of the trial, there are broad and inclusive inclusion criteria for both clinicians and patients.

#### Clinicians

Eligible clinicians are any person working as a mental health or healthcare professional within the selected NHS Trust sites (e.g., support workers, mental health nurses, occupational therapists, psychiatrists), have at least 6 months experience of working in a healthcare setting, regularly see their patients on at least a monthly basis, have experience of treating those with chronic depression and have no plans to leave their post within the next 6 months.

Clinicians are excluded if they have previous experience of using DIALOG+ or if they cannot identify at least 4 eligible patients on their caseload at the time of consenting to the study.

#### Patients

Patients are eligible if they are between 18 and 100 years old; are currently exhibiting symptoms of depression or non-psychotic low mood with a duration of illness of at least 2 years; are currently receiving treatment from an NHS mental health service with regular contact with the same clinician; have the capacity to provide informed consent and have the ability to speak and understand English to such a degree they can engage with DIALOG+ and complete the research assessment.

Following findings from the feasibility trial that chronic depression is often poorly indexed on clinical systems, the inclusion criteria was purposefully based on clinical presentation of chronic depression symptoms as opposed to a diagnosis of chronic depression disorder (e.g. F33 or F34 on the ICD-10). The treating clinician will act as the patient identifier and will use their professional judgement and access to the patient’s medical records to decide if symptoms indicating chronic depression are present. Patients with co-morbid diagnoses such as anxiety disorders and/or emotionally unstable personality disorder are eligible for inclusion.

Additionally, patients will be required to complete two screening measures to ensure that they have both a low quality of life and adequate evidence of current depression symptoms to be eligible. Patients must have a score of less than 5 on the Manchester Short Assessment of Quality of Life (MANSA) [22] and a score of 10 or more on the Montgomery-Åsberg Depression Rating Scale (MADRS) [23].

Patients will be excluded if they have a primary diagnosis of a substance misuse problem, a diagnosis of an organic mental disorder (F00-F09), are an inpatient on a psychiatric ward at the time of recruitment or do not have regular clinical contact with a mental health professional.

### Informed consent

Informed consent will be obtained by trained researchers either in person or remotely from the individual participant, with a signed copy of the form being made available to the participant.

At the point of consent, patients will have the option to agree to one of their sessions being recorded (either video or audio recorded) in order to assess adherence to the intervention, and for patients to be invited to a one-to-one interview at the end of their intervention period to discuss their experiences.

All data will be held on NHS password-protected computers or stored on NHS premises to maintain confidentiality.

### Interventions

Both the experimental intervention (DIALOG+) and the active control intervention (TAU + DIALOG Scale) will be used as part of routine care; therefore participants will continue to receive all standard treatments as part of their care. This includes medication, referral to other psychological interventions and social prescribing interventions. There are no contraindications for any other treatment and care should continue for all participants as normal. DIALOG+ and the DIALOG scale are supported by an iOS app (DIALOG v1.9.0), which will be preloaded on to an Apple iPad tablet, and provided by the research team prior to the clinician training.

The frequency of sessions will replicate what is standard for that clinician-patient dyad, although clinicians are required to deliver the interventions at least once a month for the first 6 months of the intervention delivery period.

#### DIALOG+ (intervention arm)

DIALOG+ provides an evidence-based structure to routine clinical appointments between clinician and patient. Clinicians will therefore be instructed to conduct their routine care coordination sessions, planned with their consented patients, using the steps, scales and structure offered by the intervention. DIALOG+ consists of two main parts: (1) a patient-centred assessment whereby the clinician invites the patient to rate their satisfaction with different life domains and treatment aspects (the DIALOG scale), followed by (2) a four-step approach based on the principles of solution-focused therapy.

The DIALOG scale is a computer-mediated procedure to rate 11 areas of life. Patients are asked to rate their satisfaction with eight areas of life (mental health, physical health, job situation, accommodation, leisure activities, relationship with family/partner, friendships, personal safety) and three treatment areas (medication, practical help, and meetings with mental health professionals). Each satisfaction item is rated on a rating scale of 1–7, from ‘Totally Dissatisfied’ to ‘Totally Satisfied’. The 11 areas are presented in a fixed order, and following each question, the patient is asked to rate whether they would like more help within each area [24].

Following review of the scores across the 11 areas, which includes comparing the current ratings with the ratings obtained from any previous session, up to three of the areas that are listed on the DIALOG scale are chosen to be discussed in more detail. The four-step solution-focused approach is used to structure the discussion so as to identify patients’ resources and develop solutions to deal with the patients’ concerns. At all times the ratings on the scale are referred to in order to underpin and contextualise the discussion. Step 1, Understanding, elicits contextual information about the area under discussion and establishes what is working in that area. Step 2, Looking Forward, asks the patient to adopt a future perspective and think about the ‘best case scenario’ within that domain as well as the smallest improvement that can be made to incrementally move up the rating scale. Next, Step 3, Considering Options, invites the patient to reflect on what they and others can do to in order to improve quality of life. Finally, Step 4, Agreeing on Actions, summarises the discussion and a list of actions are created and inputted into the system. Ultimately the clinician and patient together will create an action plan, made up of individual action items for each of the discussed areas to be completed before the next session.

For a more detailed description of DIALOG+, please see [16] and the DIALOG+ website [25].

All clinicians allocated to the intervention arm will receive the standardised DIALOG+ training which was developed earlier on in the programme of research. Standardised training comprises a one-off session of 60–90 min. This is followed by a mandatory ‘top-up’ session once delivery of the intervention by the clinician has begun. For practical reasons, the training will most frequently be carried out one to one, although where timings and practicalities allow, group training sessions will be allowed. Training will be facilitated by a trained researcher or the trial manager.

Training will take place as soon as possible after randomisation and can take place either face-to-face or via a Trust approved web conferencing platform. A ‘train the trainer’ model has been created whereby a senior member of the core research team can train other unblinded researchers to conduct training with clinicians. During the training session, clinicians are taught about the developmental history of the intervention, given a practical demonstration of how to use and navigate the app using the tablet computer, informed about the evidence for its effectiveness, and shown patient and clinician testimonials of those who have experience of using it. Clinicians will also be shown training videos (commissioned by the research team), and have the opportunity to participate in a roleplay exercise. Clinicians will also be provided with the DIALOG+ manual and further reading.

Throughout the duration of the study, clinicians can contact the trainers for support at any time. In addition, clinicians will also be offered at least one hour of clinical supervision. This supervision will be project-specific (i.e. additional to routine supervision) and provided by a trained therapist.

Clinicians (or patients) in the intervention arm may decide to continue with DIALOG+ after the end of the main intervention period (i.e. the first 6 months of delivery). This will be documented and considered in the analysis of outcomes after the follow-up period.

#### Active control arm (DIALOG scale + TAU)

The active control condition includes treatment as usual plus a defined intervention that also involves the use of a tablet and an assessment of the patient’s quality of life. At the end of every routine session, clinicians in the control condition, will hand the iPad to the patient and ask them to rate their satisfaction on the 11 areas of the DIALOG scale. The ratings should be completed after every routine meeting, to control for novelty effects (i.e. presence of a tablet) and repeated quality of life assessments. Patients will complete the scale alone without any input or further discussion from the clinician.

Clinicians allocated to this group will receive a shorter training session of around 15 minutes, to introduce them to the DIALOG app and explain how they should collect the scale ratings after each routine session.

### Provisions for post-trial care

All participants at the point of finishing participation in the trial will be offered a ‘mental health resources list’ which features contact details of local organisations who offer support. Researchers will also offer all participants a ‘welfare call’ 1 week after the completion of the 12-month follow-up.

### Outcomes

The trial will collect information on a range of health, social and cost-related outcomes. The scale-based measures are all well established and have been validated for use with patients with depression. All measures used in the main trial were found to have acceptable completion rates in the feasibility trial.

Outcome measures will be completed on a standardised Case Report Form (CRF) at baseline, at the end of the first 6-month intervention block (6-month follow-up) and at the end of the intervention period (12-month follow up). A shorter assessment, containing only two outcome measures (MANSA [22] and the Beck Depression Inventory (BDI-II [26];) will also be collected at 3 months for purposes of imputation.

The primary outcome is subjective quality of life, measured on the MANSA [22].

Secondary outcomes for the trial are:

- Depression symptoms as measured via observer ratings on the Montgomery-Åsberg Depression Rating Scale (MADRS) [23] and self-reported on the BDI-II [26].
- Treatment satisfaction on the Client Satisfaction Questionnaire (CSQ-8) [27].
- Illness severity on the ‘severity of illness’ subscale on the Clinical Global Impression (CGI) Scale [28]. This is clinician-rated by the patient’s clinician.
- Capability of the general adult population measured on the ICECAP-A [29].
- Health-related quality of life measured by the EQ-5D-5L instrument (EQ-5D-5L) [30].
- Costs of health service use, prescribed medication, productivity lost, burden on family and friends, and contact with criminal justice, assessed on the Client Service Receipt Inventory (CSRI [31])
- Costs of treatments from both trial arms, and costs of supervision and training to clinicians, assessed on Health Economics Inventory Forms developed by the trial health economists.
- Additionally, there will be a ‘DIALOG+ Experience Questionnaire’ completed at 6- and 12-months by those patients allocated to the intervention arm. This is a purposefully developed measure by the trial team in collaboration with the Lived Experience Advisory Panel (LEAP) to investigate the patient experience of receiving DIALOG+ as part of routine care.

All assessments are conducted by a trained researcher on NHS premises, in the community or remotely. Researchers assessing the outcomes are blinded to the allocation of the patient.

The list of outcome measures was decided upon through consultation with the Programme Steering Committee, the LEAP and the Pragmatic Clinical Trials Unit (PCTU).

Patient participants will be paid a £20 voucher for their time when completing assessments at 6- and 12-month follow-ups.

For clinicians, sociodemographic and information about their professional background including time spent working in mental health services will be collected via questionnaires at the point of recruitment.

Data collected on paper CRFs will be entered into the online OpenClinica database by trained, blinded researchers. There will be regular data monitoring visits organised by the PCTU where prime source data and data entry into the database will be reviewed.

### Sample size

The original sample size calculation was based on data from the previous DIALOG+ trial [15]. A standardised effect size of 0.35 on the MANSA (representing a mean difference of 0.31 (SD =0.9)) is equivalent to an improvement in satisfaction ratings of at least one point (on a 7-point scale) on four out of the 11 life and treatment areas on the DIALOG scale. The effect size was chosen as such an improvement is regarded as clinically meaningful [15], and related to noticeable improvement in subjective quality of life.

To detect an effect size of 0.35 (SD = 1) on the MANSA scale, and setting power at 90% for 5% significance, the total number of patients required was 172 per group (*n*=344). After accounting for clustering based on an ICC of 0.01 (as observed within the original DIALOG+ trial relating to subjective quality of life (SQoL) as measured by the MANSA), a conservative design effect of 1.04 and allowing for a drop-out rate of 20%, a total of 448 patients were needed to be recruited to give an analysable sample of 358 (179 per group). Therefore 112 clinicians were needed to be recruited, with an average of four patients per cluster. The numbers of potentially eligible patients per clinician vary by site and team, so an estimate of four patients per clinician was used in the feasibility trial and this was found to be achievable. Cluster sizes in the feasibility trial ranged from 3 to 5.

Following analysis of the TACK feasibility trial data (*in prep*), the power calculation was revised, integrating the correlation coefficient between at baseline and final follow up, on the primary outcome (MANSA). The lower end of the 95% confidence interval for the correlation coefficient was used (0.4). All other assumptions remained the same as for the original power calculation i.e., an effect size of 0.35 (SD = 1), power set at 90% and a design effect of 1.04. The updated power calculation gave a target sample size of 376, with a projected analysable sample of 300 (150 per group) when a 20% dropout rate was accounted for.

### Recruitment

As a multi-site trial, participants will be recruited from nine NHS sites and a number of clinical settings. As reflected in the inclusion criteria, any clinical team commissioned by the secondary mental health Trusts will be eligible for inclusion, so long as they meet the requirements of the session frequency and length dictated by DIALOG+ (this includes intermediary or primary care services where there is integration and a clear link to the secondary care Trust, including Improving Access to Psychological Therapies (IAPT) services).

Researchers will actively identify eligible clinical teams and individual staff members. Researchers will attend weekly multi-disciplinary team meetings to present the research study with potentially eligible clinicians/teams. Clinical teams who work with non-psychotic patients will be specifically targeted, to increase the possibility of eligibility.

After recruiting eligible clinicians, the caseload of each clinician will be screened and eligible patients identified. Members of the clinical team will approach patients and gain assent for contact by the research team. A local researcher will determine eligibility and obtain informed consent followed by completion of the screening measures and the remainder of the baseline Case Report Form (CRF), where the patient is eligible.

### Assignment of interventions: Allocation

Randomisation will be carried out remotely via e-mail from the trial manager to an independent statistician at the PCTU. The unit of randomisation is the clinician with an allocation ratio of 1:1. Randomisation will be stratified by site in blocks of 4, ensuring balanced numbers of patients in each trial arm at each NHS Trust. The allocation sequence will be via site lists created by the independent statistician on a protected server. The resulting allocation will be emailed back to the trial manager who will then inform the unblinded researcher at the relevant site. The local unblinded researcher will then be responsible for informing the clinician of their allocation.

### Assignment of interventions: Blinding

The trial manager will be unblinded to all allocations and take overall responsibility for overseeing the randomisation process. Only the trial manager can request clusters for randomisation after liaising with the independent statistician to ensure that randomisations are recorded and the correct people informed.

Each trial site will have at least one blinded and one unblinded researcher, therefore allowing the unblinded researcher to be aware of the allocation of each cluster and to inform the clinician and arrange training, etc. All other study staff, including the principal investigators, will remain blinded.

Due to the nature of the intervention, clinicians and patients will be aware of their allocation. Patient participants will be asked to not discuss the treatment they received with researchers at the data collection time points to avoid unblinding research staff.

If a researcher is unblinded accidently, or where the unblinding of a researcher is required (i.e. a principal investigator being required to assess the seriousness of a related serious adverse event), then a note will be made on the local system so that those participants will have no further direct contact with those that have been unblinded. Where blinding cannot be maintained or is broken, researchers from the coordinating centre will be used to help support local sites and provide research capacity.

### Statistical methods

The primary outcome analysis of quality of life, as measured on the MANSA, will be conducted using a mixed effects model to adjust for clustering and including baseline level of the MANSA and NHS site as covariates, as well as key demographic variables, (that are known to affect outcome) and illness severity. The treating clinician will be fitted as a random intercept effect.

The analysis will use intention-to-treat analysis by including all patients in the arm to which they were randomised, whether or not they received the intervention and including all patients in the analysis by using multiple imputation where outcomes are missing. Results will be presented as an adjusted mean difference.

Each secondary outcome will be analysed using a mixed effects model to allow for clustering and adjusting for NHS site and baseline value of the outcome.

The statistical team will remain blinded to the allocation of clusters until the database is finalised and locked for analysis.

Subgroup analyses may be conducted post hoc as a result of the variance in intervention delivery caused by the COVID-19 pandemic (see ‘COVID-19 amendments’ section).

A full statistical analysis plan will be written before data collection is complete, signed off by the Programme Steering Committee (PSC), and will be available via the project website.

### Health economic evaluation

In the economic evaluation alongside the trial, we will measure the generic health-related quality of life of participants together with the costs of providing DIALOG+ and TAU, other health/social care and societal costs of participants over a 12-month period. We will assess the cost-effectiveness of DIALOG+ from NHS and personal social services perspectives following the intention-to-treat principle.

The resource usage data for delivering interventions and training/supervising clinicians will be collected by TACK researchers using purposefully developed health economics inventory forms. Other resource usage data will be collected from patients using a customised interview-based CSRI at baseline, 6-month and 12-month follow-ups. Costs for each resource item will be calculated as a product of the quantity of resource used and its corresponding unit cost. Cost items will be summed together and presented at patient and assessment point level.

The primary outcome for economic evaluation will be EQ-5D-5L index scores, converted to quality-adjusted life years (QALYs) using the UK EQ-5D-5L value set [30]. We will conduct descriptive analyses to compare the costs and outcomes between the two trial arms at each assessment point.

Cost-effectiveness analyses will evaluate differences between patient’s total costs and QALYs between trial arms. We will use a multiple imputation approach to handle missing data. An incremental cost-effectiveness ratio (ICER) will be calculated as the extra costs incurred to produce an extra QALY. The ICER will be compared to the thresholds for cost-effectiveness typically used by NICE in the UK, i.e. £20,000 to £30,000 [32]. Uncertainty around the estimated ICER will be presented by the cost-effectiveness plane [33] and cost-effectiveness acceptability curve [34].

In the sensitivity analysis, we will (1) conduct cost-effectiveness analyses under alternative scenarios related to implementation (e.g. different combinations of staff) to help contextualise the findings for future implementation; (2) use a wider perspective by including costs from productivity lost, family and friends support and contact with criminal justice services; and (3) analyse the data for a scenario using ICECAP-A as an alternative QALY outcome measure [29, 35]. Finally, if the intervention demonstrates effectiveness during the 12-month trial period, we will study its longer-term cost-effectiveness over a 24-month period after the baseline point.

A full health economic analysis plan will be written before data collection is complete and will be available via the project website.

### Interim analyses

No formal interim efficacy analyses have been planned for the trial data. Data completeness of outcome measures will be assessed and presented to the Data Monitoring and Ethics Committee (DMEC) every 6 months during the trial.

### Process evaluation

In parallel with the trial, an embedded process evaluation will complement the results of the cluster RCT, and will use three different sources of data to enhance the understanding of how DIALOG+ is delivered, the mechanisms of change, and identify the possible barriers to wider implementation.

(1) In-depth interviews will be conducted post-intervention with approximately 36 patients and 24 clinicians purposively sampled; (2) video and audio recordings will be taken of a sample of DIALOG+ sessions and adherence to the intervention manual will assess fidelity; and (3) routinely collected data from the DIALOG app will be extracted from the clinician’s iPad at 12 month’s post-randomisation which will give data about quality of life rating changes as well as insight into the number of sessions, length of sessions, what items were selected as needing more help, and which items were selected for further discussion.

### Process Evaluation Analysis

Patients and clinicians who agree to a post-intervention interview will have sound files transcribed and analysed using framework analysis, with analysts looking for data pertaining to the experience of receiving/delivering the study intervention.

Video and audio recordings of DIALOG+ sessions will be analysed using the DIALOG Adherence Scale (v2), to check for fidelity to the core components of the DIALOG manual and training. This will help identify key areas that are overlooked in the delivery of DIALOG+ and help to improve the training resources.

Routinely collected data will be extracted from the clinician iPads and entered onto a database where descriptive data will be presented in relation to number of sessions, length of sessions, SQoL ratings (and their variance over time), and action items set.

### Oversight and monitoring

Both a PSC and a Data Monitoring and Ethics Committee (DMEC) have been convened to provide oversight to the trial. The PSC is chaired by an independent academic clinician and the DMEC chaired by an independent statistician. Both committees meet at least every 6 months to review project progress.

### Adverse event reporting

Any serious adverse events will be recorded in a specific CRF form and their relatedness to the DIALOG+ intervention will be adjudicated by the principal investigator from the local site. All principal investigators are senior clinicians.

SAEs that are unexpected or related to the intervention will be reported to the study sponsor. Upon the event being resolved the data from the CRF will be entered onto the trial online database for reporting purposes.

Any deviations from the trial protocol made by one of the research teams will be fully documented using a protocol violation log provided by the Sponsor.

### Dissemination plans

Throughout all phases of the programme of research, the study team will disseminate information about the activities and results of the trial through social media and a project-specific website [36] in order to reach a wider public audience. When results become available, they will be disseminated through:

- Scientific publications in peer-reviewed open-access journals.
- Presentations at national and international conferences and to professional and non-professional audiences at appropriate events.
- Existing research and clinical networks, including but not limited to the World Health Organization (WHO), the NIHR, the Local Clinical Research Network, organisations involved in Quality Improvement initiatives and professional networks of the programme co-applicants.

### Ethics approval

The study has been approved by the NHS Wales Research Ethics Committee 6 (REC reference 19/WA/0160).

Any amendments to the study protocol will follow the standard operating procedure provided by the Sponsor and the PCTU. This involves initial consultation with the Sponsor and relevant parties within the PCTU followed by gaining approvals from the REC (if applicable). Once permissions have been gained, sites will be notified via email with all amended documents attached and a request to update the Investigator Site File (ISF).

Any amendments to the protocol will also be added to the clinical trial registry.

### Public and patient involvement

A Lived Experience Advisory Panel (LEAP) has worked in collaboration with the study team over the entire programme of research, including the trial. The LEAP meets regularly to receive updates on study progress and to ensure that study procedures are safe and appropriate for patient participants. The LEAP reviewed all patient facing trial documents and developed the DIALOG+ Experience Questionnaire (a bespoke measure used as part of the trial). The LEAP will play an active role in the dissemination of the trial findings.

### Impact of COVID-19 and related amendments to study protocol

As a result of the COVID-19 pandemic and the sequence of national and local lockdowns during 2020 and 2021, a number of amendments were made to the study protocol, these are outlined below.

All research recruitment and randomisation activities were suspended by the study Sponsor from 18 March 2020 to 1 September 2020. During this period, delivery of the intervention was allowed to continue (as the intervention replaced routine care) but only for those patient participants already randomised. In addition, the treatment had to be completed remotely. Clinicians were therefore offered additional support and guidance on how to use DIALOG+ (particularly the app) when working remotely. DIALOG+ was designed to be an interactive, face-to-face intervention, making use of shared visual references and the collaborative sharing of equipment. Although the delivery of DIALOG+ remotely was sub-optimal- comparative to what was originally envisaged- it was decided by the clinical leads that the potential harm of stopping delivery abruptly was a higher risk than that of delivering DIALOG+ in this way. Many aspects of the intervention could continue, such as the rating of the DIALOG scale, the focus on structuring of sessions using the principles of solution-focused therapy and setting personalised action items aiming to improve satisfaction.

Originally all consent obtaining and data collection procedures (at all timepoints) were due to be conducted face-to-face by a trained researcher. In response to social distancing policies and the need for many researchers to work from home, permissions were gained for consent and study data to be collected remotely. Standard operating procedures were developed in collaboration with the study sponsor and the PCTU to ensure that this was completed in a safe and ethical manner.

All safeguarding procedures were developed in collaboration with LEAP to ensure that remote collection of sensitive data was not harmful to patient participants and the team implemented strategies such as welfare checks one week after data collection, and localised mental health resources lists to help support patients.

In parallel to the suspension of research activities, many mental health teams, especially those working in the community, were required to stop seeing patients face-to-face, either in clinic or through home visits. In the first UK lockdown from March to June 2020, many mental health services were restructured, staff seconded, or recovery teams disbanded completely. This led to high levels of dropout of recruited clinicians, and wide-ranging discharges of patient participants from services meaning they could no longer continue on in the trial.

Following guidance from the NIHR, the trial was able to restart in September 2020. However, recruitment and data collection procedures continued to be conducted remotely for the full recruitment period duration which led to long-lasting disruption to recruitment and follow-up rates. The recruitment period was originally projected to last for 12 months but this has since been extended to 30 months.

To adjust for any COVID-19 pandemic effects on the intervention itself, the outcomes or both, a sensitivity analysis may be conducted as part of the statistical analysis that will adopt a mixed-effects model approach, grouping different delivery formats of the intervention (i.e. face to face vs remote delivery vs a mixture of both).

## Discussion

At present, large numbers of patients with chronic depression regularly meet clinicians in secondary mental health settings, but these sessions are not guided by evidence-based principles. DIALOG+ is the only intervention specifically developed to make routine patient-clinician meetings in mental health care therapeutically effective. Early evidence from global work [18] has shown promising results for DIALOG+ when applied to episodic depression, but a definitive trial is required to see if a generic tool like DIALOG+ can be used on complex and long-term depression.

DIALOG+ does not require the creation of new specialist services or the restructuring of organisations, but rather can take the time and talent of existing staff to benefit the thousands of patients with chronic depression who require tailored and evidence-based support. Through the structuring of routine sessions, following the DIALOG+ manualised framework for people with chronic depression, this intervention may be a cost-saving and easily implemented way of improving quality of life, and other clinical outcomes, for this patient group.

The procedure of DIALOG+ also provides regular and consistent outcome data, i.e. patient ratings of satisfaction with life and treatment. This data cannot only be used to evaluate services on a local, regional and national level, but due to the timing of the trial can also provide an insight into how individual and group SQoL scores were impacted by the COVID-19 pandemic and the subsequent public health measures, which have been shown to have a major effect on mental health, particularly depression [2].

DIALOG+ is an existing generic and widely applicable intervention, which has been shown to be effective and implementable in a number of different clinical settings and countries. If this definitive trial shows DIALOG+ to be effective in improving outcomes for people with chronic depression then it can strengthen the implementation work already happening both nationally and globally, ensuring that patients with complex, co-morbid and chronic mental health problems can benefit from DIALOG+.

### Trial status

The trial has now completed recruitment and is in the follow-up stage. Recruitment began on the 24th of June 2019 and ended on the 28th of February 2022. The latest version of the trial protocol is v9.0, 30.Sep.2021 (available from the corresponding author on request).

## Data Availability

The final trial dataset will be available upon request to the corresponding author once all analysis is complete.

## Abbreviations

BDI: Beck Depression Inventory
COVID-19: Coronavirus disease 2019
CMHT: Community Mental Health Team
CRF: Case Report Form
CSRI: Client Services Receipt Inventory
DMEC: Data Monitoring and Ethics Committee
ELFT: East London NHS Foundation Trust
IAPT: Improving Access to Psychological Therapies
ICER: Incremental cost-effectiveness ratio
LMICs: Lower middle-income countries
MADRS: Montgomery-Åsberg Depression Rating Scale
MANSA: Manchester Short Assessment of Quality of Life
NHS: National Health Service
NIHR: National Institute for Health Research
PCTU: Pragmatic Clinical Trials Unit
QALYs: Quality-adjusted life years
RCT: Randomised controlled trial
SQoL: Subjective Quality of Life
TAU: Treatment as usual
WHO: World Health Organization
